# Supplementary material for: Serotonin 5-HT1A receptor binding and self-transcendence in healthy control subjects—a replication study using Bayesian hypothesis testing
Source: PeerJ. 2018 Nov 16;6:e5790. doi: 10.7717/peerj.5790 (PMC6241390; doi:10.7717/peerj.5790)
Supplement: Supplemental Information 1 [file peerj-06-5790-s001.pdf]

Supplementary information

Results after twin exclusion and using cerebellar white matter as reference region

Table 3 *Pearson's r and default BF for 5-HT_{1A} receptor binding and self-transcendence (ST) and spiritual acceptance (SA) assessed by TCI for frontal cortex, hippocampus and dorsal raphe nuclei for present replication and Karlsson's replication.*

	Pearson's r			BF_{0-}		
		Twin exclusion	White matter		Twin exclusion	White matter
**Self-transcendence**						
- frontal cortex	-0.06	0.16	-0.07	5.3	8.3	3.7
- hippocampus	-0.19	-0.02	-0.23	2.5	4.3	5.9
- dorsal raphe nuclei	-0.11	-0.10		4.3	2.9	
**Spiritual acceptance vs material rationalism**						
- frontal cortex	-0.03	0.19	-0.01	5.6	9.1	5.3
- hippocampus	-0.12	0.07	-0.13	4.2	6.3	2.4
- dorsal raphe nuclei	-0.16	-0.09		3.1	2.9	

Abbreviations: TCI, Temperament and Character Inventory; r, Pearson's correlation efficient; BF_{0-}, the default BF representing the likelihood for the null hypothesis (H_0: no correlation) versus the alternative hypothesis (H_-: negative correlation).

Calculation of PPV

PPV is the positive predictive value (i.e. the chance of the finding being true) and has the following formula:

$$PPV = \frac{(1-\beta)\times R}{(1-\beta)\times R+\alpha}$$

where $1 - \beta$ is the power and β the type II error; R is the pre-study odds (in our case the probability of any one personality scale being correlated with 5-HT_{1A} receptor binding); α is the type I error (the statistical significance level).

We made the following assumptions:

- We calculate for an effect size of Pearson's R = 0.5. This represents a large effect (Cohen, 1988). We calculated the power using this effect size using the "pwr" package in R (Stephane Champely (2015).
- We assume a pre-study odds of 10%. We believe that this is a liberal estimate for comparisons of receptor binding and personality traits.
- The sample size of the original study was 15.
- We set alpha = 0.05 for 10 analyses without correction for multiple comparisons. The original analysis was performed first with three ROIs for seven dimensions, after which self-transcendence was found to be significantly associated with ST ($p < 0.05$). Due to the high correlations between regional binding estimates, it is an exaggeration to consider this as 21 independent comparisons. Instead, we approximate it as 10 comparisons, comprised of 7 approximately independent original personality dimensions, and three more to account for the multiple ROIs. This means that we assume a final type I error rate of 0.5 (50%).
